# Frovatriptan vs other triptans in the treatment of menstrual migraine: pooled analysis of three double-blind, randomized, cross-over studies

**DOI:** 10.1186/1129-2377-14-S1-P191

**Published:** 2013-02-21

**Authors:** G Allais, V Tullo, S Omboni, C Benedetto, G Sances, D Zava, MD Ferrari, G Bussone

**Affiliations:** 1 Department of Gynecology and Obstetrics, Women's Headache Center, University of Torino, Torino, Italy; 2 Department of Clinical Neurosciences, Carlo Besta National Neurological Institute, Milano, Italy; 3 Italian Institute of Telemedicine, Varese, Italy; 4 Headache Centre, IRCCS C. Mondino Foundation, Institute of Neurology, Pavia, Italy; 5 Istituto Lusofarmaco d’Italia, Milano, Italy; 6 Leiden Centre for Translational Neuroscience, Department of Neurology, Leiden University Medical Centre, Netherlands

## Objective

To review the efficacy and safety of frovatriptan (F) vs. rizatriptan (R), zolmitriptan (Z) and almotriptan (A), in women with menstrually related migraine (IHS criteria) through a pooled analysis of three individual studies.

## Methods

Subjects with a history of migraine with or without aura were randomized to F 2.5 mg or R 10 mg (study 1), F or Z 2.5 mg (study 2), and F or A 12.5 mg (study 3). The studies had an identical multicenter, randomized, double blind, cross-over design. After treating 3 episodes of migraine in no more than 3 months with the first treatment, patients had to switch to the next treatment for other 3 months.

## Results

346 subjects formed the main study intention-to-treat population; 280 of them were of a female gender (81%) and 236 in the fertile age. A total of 187 out of the 236 eligible women (79%) treated at least one episode of menstrual migraine with both medications and were thus included in the present subgroup analysis. Rate of pain free at 2, 4 and 24h was 23%, 52% and 67% with F and 30%, 61% and 66% with comparators (p=NS). Pain relief episodes at 2, 4 and 24h were 37%, 60% and 66% for F and 43%, 55% and 61% for comparators (p=NS). Rate of recurrence was significantly (p<0.05) lower under F either at 24h (11% vs. 24% comparators) or at 48h (15% vs. 26% comparators). Number of menstrual migraine attacks associated with drug-related adverse events was equally low (p=NS) between F (5%) and comparators (4%).

## Conclusions

According to our analysis of individual studies F is as effective as other triptans in the immediate treatment of menstrual migraine attacks, but exhibits a more sustained effect.
